# Cost and Cost-Effectiveness of a Demand Creation Intervention to Increase Uptake of Voluntary Medical Male Circumcision in Tanzania: Spending More to Spend Less

**DOI:** 10.1097/QAI.0000000000001682

**Published:** 2018-03-19

**Authors:** Sergio Torres-Rueda, Mwita Wambura, Helen A. Weiss, Marya Plotkin, Katharine Kripke, Joseph Chilongani, Hally Mahler, Evodius Kuringe, Maende Makokha, Augustino Hellar, Carl Schutte, Kokuhumbya J. Kazaura, Daimon Simbeye, Gerry Mshana, Natasha Larke, Gissenge Lija, John Changalucha, Anna Vassall, Richard Hayes, Jonathan M. Grund, Fern Terris-Prestholt

**Affiliations:** * Department of Global Health and Development, London School of Hygiene & Tropical Medicine, London, United Kingdom;; † National Institute for Medical Research (NIMR), Mwanza, Tanzania;; ‡ MRC Tropical Epidemiology Group, London School of Hygiene & Tropical Medicine, London, United Kingdom;; § Jhpiego Tanzania, Dar es Salaam, Tanzania;; ║ Currently, Jhpiego, Baltimore, MD;; ¶ Avenir Health, Project SOAR, Washington, DC;; # Currently, District Commissioner's Office, Meatu, Simiyu, Tanzania;; ** Current, FHI360, Washington, DC;; †† Strategic Development Consultants, Durban, South Africa;; ‡‡ Centers for Disease Control and Prevention, Center for Global Health, Division of Global HIV & TB, Dar es Salaam, Tanzania;; §§ Ministry of Health and Social Welfare, National AIDS Control Program, Dar es Salaam, Tanzania;; ║║ Centers for Disease Control and Prevention, Center for Global Health, Division of Global HIV & TB, Atlanta, GA; and; ¶¶ Currently, Centers for Disease Control and Prevention, Center for Global Health, Division of Global HIV & TB, Pretoria, South Africa.

**Keywords:** VMMC, HIV prevention, cost-effectiveness, economic evaluation

## Abstract

**Background:**

Although voluntary medical male circumcision (VMMC) reduces the risk of HIV acquisition, demand for services is lower among men in most at-risk age groups (ages 20–34 years). A randomized controlled trial was conducted to assess the effectiveness of locally-tailored demand creation activities (including mass media, community mobilization, and targeted service delivery) in increasing uptake of campaign-delivered VMMC among men aged 20–34 years. We conducted an economic evaluation to understand the intervention's cost and cost-effectiveness.

**Setting:**

Tanzania (Njombe and Tabora regions).

**Methods:**

Cost data were collected on surgery, demand creation activities, and monitoring and supervision related to VMMC implementation across clusters in both trial arms, as well as start-up activities for the intervention arms. The Decision Makers' Program Planning Tool was used to estimate the number of HIV infections averted and related cost savings, given the total VMMCs per cluster. Disability-adjusted life years were calculated and used to estimate incremental cost-effectiveness ratios.

**Results:**

Client load was higher in the intervention arms than in the control arms: 4394 vs. 2901 in Tabora and 1797 vs. 1025 in Njombe, respectively. Despite additional costs of tailored demand creation, demand increased more than proportionally: mean costs per VMMC in the intervention arms were $62 in Tabora and $130 in Njombe, and in the control arms $70 and $191, respectively. More infections were averted in the intervention arm than in the control arm in Tabora (123 vs. 67, respectively) and in Njombe (164 vs. 102, respectively). The intervention dominated the control because it was both less costly and more effective. Cost savings were observed in both regions stemming from the antiretroviral treatment costs averted as a result of the VMMCs performed.

**Conclusions:**

Spending more to address local preferences as a way to increase uptake of VMMC can be cost-saving.

## Abstract 2

Supplemental Digital Content is Available in the Text.

## INTRODUCTION

Male circumcision has been demonstrated to reduce the risk of male acquisition of HIV.^1–3^ Following the WHO and UNAIDS recommendations that voluntary medical male circumcision (VMMC) be scaled up in settings where it would have the highest impact,^4^ 14 countries in sub-Saharan Africa have rolled out VMMC, with an estimated 11.7 million men circumcised through 2016.^5^ Tanzania incorporated VMMC into its national HIV prevention strategy in 2009, with a goal of circumcising 2.2 million males aged 10–34 years by 2017^6^; as of September 2016, more than 1.6 million VMMCs had been performed in the country.^5^ Although the priority population outlined in the national strategy is men aged 10–34 years, modelling data suggest that circumcising men aged 20–34 years would yield a more immediate impact on HIV reduction.^7–9^

Studies have examined the cost and cost-effectiveness of VMMC in sub-Saharan Africa. Estimates of the average cost per surgical VMMC range from $29 in sites integrated into regular health facility activities in Kenya^10^ to $158 in public hospitals in South Africa.^11^ Research indicates that lower unit costs are observed in fixed delivery sites compared with outreach or mobile sites and that delivering VMMC in health centers is less costly than hospital-based delivery.^10–13^ Staff and consumable supplies are the 2 largest cost drivers across delivery modalities, with capital costs also being substantial in mobile delivery sites.^13,14^ Research on target regions suggests that rapid scale-up of VMMC would require substantial additional investment, particularly in the early stages, but could lead to high long-term savings if human resources constraints are properly addressed.^13,15,16^

Studies modelling the cost-effectiveness of VMMC in terms of cost per HIV infection averted^9,10,13,16–26^ have shown wide differences: from $78 in Kenya^10^ to $22,000 in Rwanda.^26^ Key factors underlying this variation include VMMC unit cost, projected protective effect of VMMC, HIV prevalence and incidence, time horizons considered, and VMMC coverage. A limited number of studies have looked at the cost per disability-adjusted life years (DALYs) averted. These studies have suggested that VMMC is cost-effective (with cost per DALY averted ranging from $7 to $120), and once incremental cost-effectiveness ratios (ICERs) were calculated including antiretroviral treatment (ART) costs averted, VMMC seems to be cost-saving when compared with a no-VMMC scenario.^15,20,24,27^

Models have also suggested that programs can be more cost-effective by targeting men in most at-risk age groups. In Zimbabwe, the modelled cost per HIV infection averted dropped from $1035 when assuming the current age distribution (13–29 years) to $811 when only men aged 20–24 years were circumcised.^17^ Recent studies in Swaziland, Malawi, Uganda, South Africa, and Tanzania suggest that the most cost-effective age group to target is men aged 15–34 years.^9,22–25^

These age-targeted models, however, have recognized limitations: they are not based on empirical data that account for variations in cost stemming from different demand creation and service delivery approaches, which may be necessary to increase uptake of VMMC services for those at highest risk. Previous studies have documented the barriers to client demand for VMMC.^28–32^ Lower levels of health-seeking behavior in men, fear of pain and injections, perceptions of inconvenience with an outcome of partial protection against HIV, and minimal awareness of the health benefits of circumcision have been suggested as possible reasons for lower VMMC uptake. Locally-tailored demand creation approaches have been proposed as a way to overcome these challenges.^33^

As far as we are aware, no articles have been published either (1) presenting primary cost data on the resources needed to design and implement locally-tailored demand creation approaches or (2) analyzing the outcomes resulting from these additional costs in terms of HIV infections and DALYs averted, which would allow policymakers to weigh the effect of additional investments against other HIV prevention strategies, and, more broadly, investments in other disease areas. This study seeks to bridge that gap in the literature by analyzing the cost and cost-effectiveness of a targeted demand creation intervention in the context of a randomized controlled trial in Tanzania evaluating VMMC uptake among men aged 20–34 years in 2 settings with different HIV-prevalence rates.^34^

## METHODS

### Setting and Trial

A randomized controlled trial was conducted in Njombe and Tabora regions of Tanzania in November–December 2014 and February–March 2015, respectively. By the start of the intervention, VMMC coverage across age groups was higher in Njombe (80%) than in Tabora (55%)^35^ but the absolute number of men aged 20–34 years was higher in Tabora than in Njombe^36^ (see Supplemental Digital Content 1, http://links.lww.com/QAI/B160 for details). HIV prevalence was higher in Njombe (14.8%) than in Tabora (5.1%).^37,38^ It is important to note that the intervention was conducted at different points of the agricultural cycle in the 2 regions: during the dry season in Tabora and rainy season in Njombe.

The trial evaluated locally-tailored demand creation and delivery of VMMC services. In addition to all the standard demand creation components present in the control arms (which included elements of mass media engagement, community mobilization, and targeted service delivery), the following were added to the intervention arms: (1) demand creation communication stressing non-HIV benefits of VMMC and the voluntary nature of HIV testing services before VMMC; (2) additional peer promoters and the involvement of 2 circumcised men from the community as auxiliary peer promoters; (3) separate waiting and group education areas for men aged ≥20 years during service delivery; (4) engagement of female partners in community-based demand creation and education about post-circumcision healing and abstinence (see Supplemental Digital Content 2 for details on demand creation activities in both arms, http://links.lww.com/QAI/B160).

Details of the trial design and outcomes have been published.^34^ The trial aimed to assess VMMC uptake among men aged 20–34 years following the locally-tailored demand creation strategy described compared with the standard of care normally delivered. Ten clusters were randomly selected per region and evenly allocated across control and intervention arms. Within each cluster, VMMC started in a “parent” VMMC site. If the number of VMMCs fell below a predetermined threshold, a “spin-off” VMMC site opened elsewhere within the cluster.

Parent sites and spin-off sites received demand creation activities (for 2 weeks and 1 week, respectively) before surgical services were offered. VMMC services were delivered for 26–34 days in each parent site. Clinical staff remained in the cluster for the duration of the trial and did not perform any other clinical services other than the VMMCs. Sites stayed open for follow-up visits after circumcisions ended. Surgeries were conducted using a mixture of reusable and disposable kits.

### Costing Methods

Costs of all activities related to surgery (which includes HIV testing services and waste management), demand creation, and monitoring and supervision for the control and intervention arms of the trial were included. Costs of start-up activities for the intervention arms, namely orientation meetings for peer promoters and auxiliary peer promoters and the development of demand creation materials (which includes formative research to determine user preferences), were included in the analysis. Start-up activities for the standard of care delivery and surgery training were excluded; these activities took place long before the start of the intervention and cost data were not available.

The study adopted a provider's perspective (ie, costs of intervention delivery). A top–down costing approach (ie, dividing overall program costs by outputs) was used to account for inefficiencies, down time, and wastage. Limitations of this approach (a lack of information on individual service provision and an over reliance on implementing organization records) are acknowledged.

Staff, supplies, and capital costs (ie, goods with a market value of ≥$100 and useful life of more than 1 year) for surgery and demand creation activities were calculated using cluster-specific data. Total monitoring and supervision costs and start-up costs (in the intervention arms) were calculated and allocated across clusters in both arms. Costs of developing demand creation materials were annuatized over 5 years. Financial records of the implementing organization and the Central Medical Stores catalog were used to calculate prices of supplies and capital goods.^39^ Useful life years of reusable and capital goods were estimated by interviews with the implementing organization.

The number of days worked by clinical and demand creation staff was ascertained by attendance record review. Supervision and start-up staff time was determined through interviews with the implementing organization. Salary costs were obtained from health care providers who conducted the VMMCs and averaged by clinical category. Demand creation staff were paid per diems at the standard rate of the implementing organization. Supervision and start-up salary information was gathered from interviews and record review with the implementing organization.

Costs per cluster were estimated and unit costs (average costs per VMMC) were calculated by dividing total cluster costs by the total number of circumcisions performed per cluster. Cost drivers were explored by calculating average cluster cost components in each trial arm per region (ie, total cost components across all cluster per arm/region divided by the total number of clusters in each arm/region).

Tests of statistical significance to examine cost differences between arms were not performed because of the low number of clusters per region. Cost data were collected in Tanzanian shillings (TSH) and converted to 2015 U.S. dollars using an average rate of 2130 TSH per $1.^40^

### Effectiveness Analysis

Data on the number, client age, and location of VMMCs performed were obtained from the trial data set.^34^ Trial results showed that the total number of VMMC clients of all ages was higher in the intervention arms than in the control arms: 6251 vs. 3968, respectively (RR 1.4). Although the total number and proportion of clients aged 20–34 years were greater in the intervention arms, there was a non-significant difference in the proportion of men aged 20–34 years between intervention and control arms (11.3% vs. 14.7%, respectively) in Njombe, whereas a two-fold statistically-significant difference was observed in Tabora (27.5% vs. 11.5%).^34^ See Supplemental Digital Content 3, http://links.lww.com/QAI/B160 for a breakdown of the number of VMMC clients per age group, region, and trial arm.

The Decision Makers' Program Planning Tool (DMPPT) version 2.1 was used to estimate the primary number of HIV infections averted over a 15-year time horizon from the total number of VMMCs performed during the trial, as well as the costs saved from ART averted.^26,41^ Details on the calibrations and methods used in the Tool have been published.^26,41^ The DMPPT used country- and region-specific demographic data, epidemiological dynamics, and ART costs^1–3,9^ and estimated the differential impact of VMMC by client age group. Therefore, effectiveness estimates take into account the age distributions of clients circumcised in the trial.

DALYs averted following HIV infections averted as a result of VMMC were calculated using a standard approach.^42^ Life expectancy was set at 69 years^43^ for both HIV-negative people and HIV-positive people on ART. ART coverage in Tanzania was assumed to be 70%,^44^ and disability weights applied allowed for ART access variation. Annual cost of ART (including antiretroviral medications, related laboratory costs, staff costs, and overheads) was assumed to be $515 per patient. Averting an HIV infection would avert 21.2 DALYs for an individual without ART access, whereas just 3.26 DALYs would be averted among an individual using ART. A standard 3% discount rate was applied to costs, and HIV infections and DALYs averted. Supplemental Digital Content 4, http://links.lww.com/QAI/B160, summarizes input variables used to calculate infections and DALYs averted.^1–3,9,26,41,43–48^

### Cost-Effectiveness and Sensitivity Analysis

The primary outcome of analysis is the ICER estimated by comparing cost per DALY averted using a tailored demand creation approach against cost per DALY averted using the standard of care approach. Intermediate measures are total cost per cluster and unit cost per VMMC, per HIV infection averted, and per DALY averted.


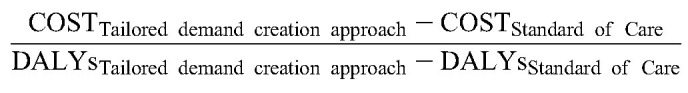


A univariate deterministic sensitivity analysis was performed to measure the effect of uncertainty on a number of different parameters (including ART coverage and cost, time horizon, and supplies costs) on the ICERs.

Ethical approval was obtained from the London School of Hygiene & Tropical Medicine Research Ethics Committee, the Medical Research Coordinating Committee of the National Institute for Medical Research of Tanzania, and the Institutional Review Board of the U.S. Centers for Disease Control and Prevention. The study was also registered at ClinicalTrials.gov (NCT02376348).

## RESULTS

Average total costs per cluster were higher in the intervention arms than in the control arms both in Njombe ($46,696 and $39,105, respectively) and in Tabora ($54,408 and $40,443, respectively). The breakdown by cost category (as percentage of total cost) was similar between control and intervention arms, with recurrent staff costs accounting for the largest portion of total costs (between 68% and 80%). The majority of these staff costs were related to surgery. Demand creation–related staff costs were more than 2 times higher in the intervention arms than in the control arms ($6577 vs. $2438, respectively) (Table 1).

**TABLE 1.**
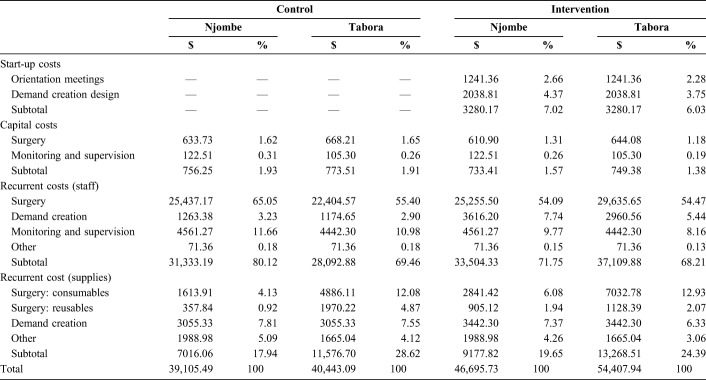
Breakdown of Average Cluster Costs by Region and Arm (USD and %)

Given the VMMC delivery model implemented, most costs across both arms of the trial can be considered cluster-level fixed costs (ie, costs that remain constant at the cluster level independent of outcome). Only consumable surgery supplies can be considered variable costs (ie, costs that vary with output), and they accounted for 4% to 6% of the total costs in Njombe and 12% to 13% in Tabora.

**FIGURE 1.**
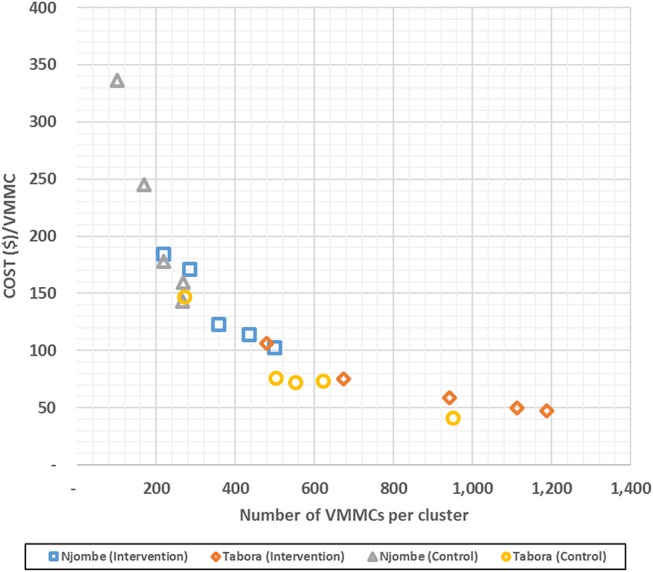
Plot graph of average cost per VMMC per cluster. The graph shows the average cost per VMMC per cluster (unit cost) plotted against the number of VMMCs per cluster. The data suggest that unit costs decrease as the number of VMMCs per cluster increases.

Average costs per VMMC per cluster varied greatly, between $40.86 and $336.66. Average costs per VMMC per arm were lower in the intervention clusters than in the control clusters: $81.65 vs. $101.31, respectively. They were also, on average across both arms, more than 2 times lower in Tabora than in Njombe: $65.01 vs. $152.02, respectively. Cluster-specific unit costs and total VMMCs can be found in Supplemental Digital Content 5, http://links.lww.com/QAI/B160. An inverse relation between the number of VMMCs performed per cluster and unit costs per cluster can be observed: the more VMMCs performed, the lower the unit cost (Fig. 1).

The impact of VMMC on HIV infections averted over 15 years would be greater in the intervention arm than in the control arm across the 2 regions: 287 infections would be averted compared with 169, respectively (Table 2). Although the total number of VMMCs was higher in Tabora than in Njombe, the impact on HIV infections averted was projected to be higher in Njombe than in Tabora, with 266 infections averted compared with 190, respectively, because of higher projected HIV incidence in Njombe. The costs per HIV infection averted were lower in the intervention arm than in the control arm both in Njombe ($1424 vs. $1917) and in Tabora ($2212 vs. $3018).

**TABLE 2.**
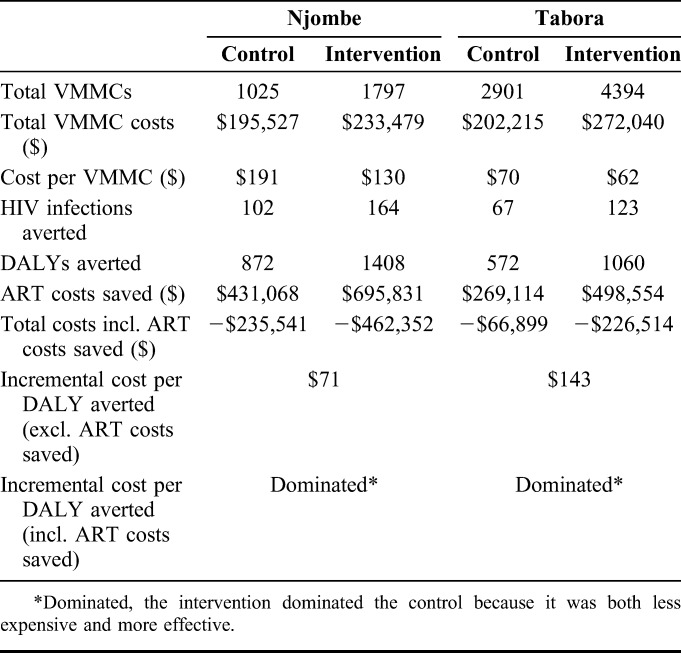
Effectiveness: HIV Infections Averted, DALYs Averted, and Costs

Similar trends emerged in DALYs averted (Table 2). In total, more DALYs were averted in the intervention arms (2468) than in the control arms (1444). Again, despite fewer total VMMCs in Njombe than in Tabora, 2280 DALYs were averted in Njombe and 1632 in Tabora. The costs per DALY averted were lower in the intervention arm than in the control arm in Njombe ($166 vs. $224) and in Tabora ($257 vs. $354).

**FIGURE 2.**
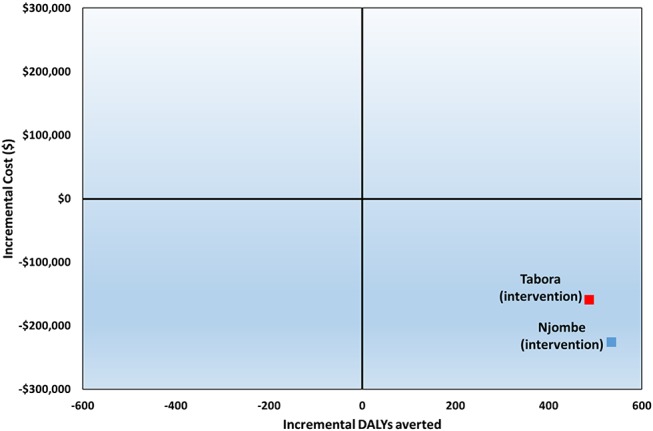
Incremental cost-effectiveness plane. The 2 squares denote the ICERs for both study regions. Their position in the South-East quadrant suggests that the intervention dominates the control because it is both less costly and more effective.

The ICERs of DALYs averted (without including cost savings from ART costs avoided from clients who did not acquire HIV) were $71 in Njombe and $143 in Tabora. However, when ART costs averted were considered, cost savings were achieved resulting in negative ICERs in both regions (Fig. 2).

**FIGURE 3.**
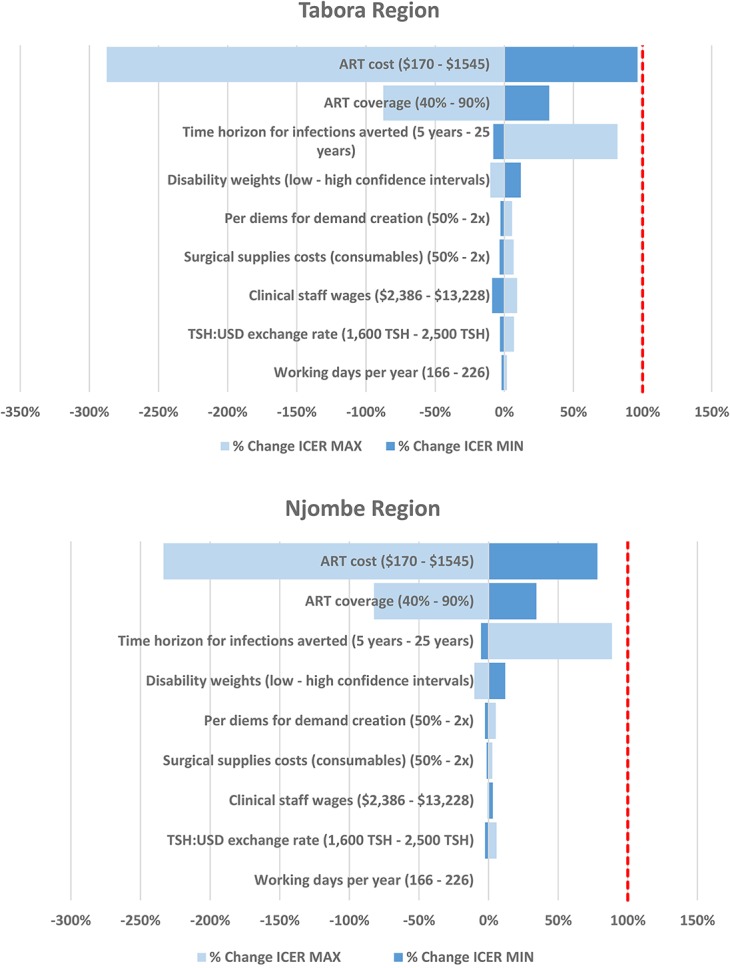
Tornado diagrams of the percentage changes in the base–case ICER (including averted costs of ART) from a deterministic 1-way analysis of key input variables per region. (1) Light blue bars show the direction and magnitude of change of the ICER when the input is at its minimum value. Conversely, dark blue bars show the direction and magnitude of change of the ICER when the input is at its maximum value. (2) Base case assumptions of parameters: ART costs: $515; ART coverage: 70%; time horizon for infections averted: 15 years; disability weights: HIV symptomatic pre-AIDS 0.274 (0.184–0.377), HIV/AIDS receiving ART 0.078 (0.052–0.111); AIDS: not receiving ART 0.582 (0.406–0.743); per diems demand creation: peer promoter $21 USD, auxiliary peer promoter $11 USD; surgical supplies costs (consumables): Tabora $24,431 (control) and $35,164 (intervention), Njombe $8070 (control) and $14,207 (intervention); clinical staff wages: $7938 USD; TSH to US dollar exchange rate: 2130 TSH:$1 USD; working days per year: 191. (3) The dotted line indicates the point at which the ICER goes from being cost-saving (left) to not cost-saving (right).

Univariate sensitivity analysis suggests that the ICERs were relatively robust to variations in VMMC cost inputs. The greatest impact on the ICERs was driven by the cost of ART. ICERs remained negative in all variations tested, indicating that the demand creation intervention would remain cost-saving under most scenarios. Modifying the yearly cost of ART between $175 and $1545 (one-third to 3 times the base cost) led to variations in the base–case ICERs from −233% to 78% in Njombe and from −287% to 96% in Tabora. The variables examined held the same hierarchy in both regions in terms of relative sensitivity (Fig. 3).

## DISCUSSION

To our knowledge, this is the first economic evaluation examining a locally-tailored demand creation strategy to improve uptake of VMMC among target populations.

Although higher costs associated with the demand creation activities were incurred in the intervention arms, the unit costs per VMMC were lower in the intervention arms because of increased uptake. Despite higher unit costs and lack of impact of the intervention on the proportion of clients in the 20–34 year age group in Njombe, costs per infection averted and per DALY averted were lower due to higher projected HIV incidence. When taking into account the costs of ART provision avoided because of HIV cases averted, providing VMMCs was cost-saving in both arms of the trial and across the 2 regions. However, the intervention arms were more cost-saving than the control arms. Staff costs made up the largest proportion of costs in both arms of the trial, followed by supplies. Although this is consistent with other studies, staff costs as a percentage of total cost were higher in our study than in others.^13,49^ This is likely due to the service delivery strategy used. As expected, the cost of the demand creation activities as a proportion of total cost was higher in the intervention arms than in the control arms; the main incremental costs associated with the tailored demand creation were 2 extra peer promoters per intervention cluster and 2 auxiliary promoters. Despite the added demand creation costs, these activities increased the total number of clients in both regions and the proportion of clients aged 20–34 years in Tabora.

Most other studies used ingredients-based costing, which only capture direct time spent providing VMMC services. This study used a top–down costing approach, which accounts for all staff costs, whether or not the staff were fully utilized; this may explain our relatively higher staff costs. The service delivery approach used in the trial (in both arms) required all VMMC staff to remain in the same cluster for a minimum of 4 weeks regardless of client volume and to not perform other clinical activities. In routine service delivery (ie, non-research), staff move frequently, as dictated by client volume. Therefore, staff costs in our study can be considered to be fixed costs. One other published study had a similar delivery method to ours, although in their case the mobile clinics remained in the field for fewer days (12 on average vs. approximately 30 in our study).^12^

Although these higher costs related to fixed clinical staff could be interpreted as an inefficiency, this approach may be an essential component of an effective strategy in delivering services to harder-to-reach populations. Whether staff-heavy modalities of delivery are essential to an increased uptake of VMMC among harder-to-reach clients needs to be assessed and piloted in routine service delivery settings.

Our findings suggest that economies of scale were achieved. Figure 1 shows that the higher the number of VMMCs per cluster, the lower the unit costs, likely because of the high proportion of cluster-level fixed costs (between 87% and 96%) across both arms and regions. Although it is beyond the scope of this study to explore economies of scale in detail (and further work in this area, such as cost function analyses, would be a worthwhile endeavor), our study suggests that once the initial costs of setting up a cluster are incurred, a lower cost per VMMC can be attained if demand creation activities succeed in attracting more uncircumcised men.

The average cost per HIV infection averted ranged from $1424 in the intervention arm in Njombe to $3018 in the control arm in Tabora. These costs were within the ranges found in other studies, although the range is extremely wide. The locally-tailored demand creation strategy in the intervention arms required additional start-up costs (orientation meetings and a demand creation design stage, including formative research), as well as additional demand creation staff (peer promoters and auxiliary peer promoters) and additional demand creation supplies (ie, more flyers and banners, etc.). However, the average cost per HIV infection averted and cost per DALY averted were lower in the intervention arms than in the control arms in both regions. This suggests that additional spending on demand creation, which in turn may lead to higher demand, is justified as it makes the intervention more cost-effective than the standard of care.

Although average unit costs per VMMC were higher in Njombe for both the control and intervention arms, the average costs per HIV infection averted and per DALY averted were lower in Njombe. Fewer people were circumcised during the trial in Njombe, but the impact per circumcision on HIV acquisition was projected to be greater in that region because of higher projected HIV incidence.

Total costs of the intervention decreased when incorporating cost savings attributable to ART averted. The ICERs for both regions were negative: the intervention dominated the control as the intervention was both less expensive and more effective. We therefore found that the intervention was not only cost-effective but was also cost-saving. Other studies have also found that VMMC is cost-saving when compared with a no-VMMC scenario once accounting for ART costs averted.^15,20,24,27^ However, our findings are novel because they show that the locally-tailored demand creation approach in the intervention is cost-saving, not only in relation to the no-VMMC scenario, but also to the standard of care approach.

It is important to note, however, that as coverage of VMMC increases in the future, greater efforts will need to be made to reach an increasingly small number of uncircumcised men. Demand creation strategies may need to be strengthened or modified, incurring additional cost. These changes could potentially make the intervention less cost-effective.

Although the intervention increased overall VMMC uptake, the proportion of men in the target age range did not increase significantly in Njombe. Despite this, we found that, even if cost savings were observed in both regions, impact and cost-effectiveness in Tabora were lower than in Njombe. This suggests that the overall projected HIV incidence may be more important than client age in determining cost-effectiveness of VMMC.

### Limitations

We may have underestimated the cost-effectiveness (DALYs averted) of the intervention for several reasons: (1) although VMMC reduces the risk of acquiring other infections,^50^ our study considers only the costs related to averting HIV infections; (2) the DMPPT model is not dynamic and thus the number of secondary HIV infections averted are underestimated; and (3) the trial design required that staff stay in 1 cluster regardless of the number of daily clients, whereas in a nonstudy setting, staff would relocate according to client volume, meaning our staff costs are likely higher when compared with other services using outreach models. Conversely, we may have overestimated the cost-effectiveness of the intervention by not including start-up costs for the control arms.

Patient costs were not included in this study. Given the period of recommended physical inactivity post-VMMC, the costs of wages lost to the individual may be substantial.^51^ A separate analysis of patient costs may identify their effect on VMMC uptake.

The intervention arms had several components. However, activity-specific costs and effects were not disaggregated. Future research could look at which specific components yield the greatest uptake of VMMC and the consequent cost implications.

Some of the costs in our study, such as monitoring and supervision costs, were collected for each region and could not be disaggregated at the cluster level. Therefore we allocated these costs equally across clusters in the same region, potentially underestimating the costs in clusters that required greater oversight.

Trial implementation took place during the dry season in Tabora and the rainy season in Njombe, when more agricultural activity takes place. This may have led to differences in implementation costs and demand for services among men of productive ages.

Three separate, self-contained models and tools were used (costs, DMPPT, and DALYs), and therefore, a combined probabilistic sensitivity could not be undertaken. Consequently our analysis does not capture full uncertainty.

## Supplementary Material

SUPPLEMENTARY MATERIAL
