# Supplementary material for: Cost and Cost-Effectiveness of a Demand Creation Intervention to Increase Uptake of Voluntary Medical Male Circumcision in Tanzania: Spending More to Spend Less
Source: J Acquir Immune Defic Syndr. 2018 Mar 19;78(3):291–9. doi: 10.1097/QAI.0000000000001682 (PMC6012046; doi:10.1097/QAI.0000000000001682)
Supplement: SUPPLEMENTARY MATERIAL [file qai-78-291-s001.docx]

**Supplemental Digital Content 1: Number of men in the population and percentage circumcised per age group and region (Njeuhmeli et al., 2016; Tanzania National Bureau of Statistics, 2012)**

|  | **Total number of men in the region** | | | **Percentage of men circumcised** | | |
| --- | --- | --- | --- | --- | --- | --- |
|  | 20-24 years | 25-29 years | 30-34 years | 20-24 years | 25-29 years | 30-34 years |
| **Njombe** | 26,301 | 23,572 | 20,340 | 97% | 68% | 53% |
| **Tabora** | 91,734 | 74,305 | 62,872 | 59% | 51% | 51% |

**Supplemental Digital Content 2: Components of the VMMC strategy in the control and intervention arms (**[**Wambura, Mahler et al. 2017**](#_ENREF_10)**)**

|  | **Control Arm** | **Intervention Arm** |
| --- | --- | --- |
| **Mass media** | Radio spots on local stations advertising the location and dates of VMMC services. | Same as control |
| **VMMC Community Promotion** | A vehicle with a megaphone visited communities near VMMC sites and promoted specific information about VMMC and the names of service delivery sites.  Information about VMMC was distributed prior to football matches in the communities.  Flyers promoting VMMC were distributed to adolescents, their guardians, women, and men. | Same as control  PLUS  A vehicle with a megaphone visited intervention communities and emphasized the non-HIV-related benefits of VMMC such as penile hygiene and cleanliness, and the prevention of cervical cancer among female partners of VMMC clients. Messages also included information that:   - Addressed myths about the inappropriate disposal of foreskins. - Promoted the professionalism of VMMC clinicians (male & female) to address concerns about being attended by female staff. - Provided information about separate waiting areas and group counselling for men aged 20 and above, and other measures to increase privacy in the facility. |
| **VMMC Peer Promotion** | 2 - 4 peer promoters were assigned to each outreach facility. These promoters (a mix of men and women) conducted group and individual demand creation activities in the communities, and worked with community leaders and organizations to promote services. | Same as control  PLUS  Circumcised men and female partners of circumcised men from the community) were paired with peer promoters to:   - Conduct one-on-one and small group activities in the community with potential clients aged 20-34 years. - Visit community leaders to promote VMMC services. - Prioritize the non-HIV benefits listed above in their messaging. - Man information booths for older clients and their partners on VMMC in the communities. These booths provided information, education, and /counselling sessions specifically for women wanting more information about VMMC. |
| **Facility preparation** | A half-day site preparation and the VMMC orientation session were held for the personnel who worked at the outreach site. | Same as control  PLUS  Separate waiting areas and group education areas were established for adult men at the VMMC sites. Where possible, adults were circumcised in a separate space from the younger males. Additional privacy measures were put in place at all intervention facilities (e.g. extra screens). |
| **Client package** | Clients seeking services received the full WHO package for voluntary medical male circumcision, which included group education, individual counselling, physical screening including sexually transmitted infections check and opt-out HIV testing, circumcision under local anesthesia, immediate post-operative exam, and two return visits. | Same as control  PLUS  Adult clients were specifically asked to refer their friends and encouraged to refer their female partners to the community’s information booth. |

**Supplemental Digital Content 3: VMMC clients per age group, region and trial arm**

| **Age group (years)** | **NJOMBE** | | **TABORA** | |
| --- | --- | --- | --- | --- |
|  | **Control** | **Intervention** | **Control** | **Intervention** |
| 10-14 | 583 | 1124 | 1926 | 1788 |
| 15-19 | 254 | 280 | 562 | 1071 |
| 20-24 | 103 | 110 | 208 | 744 |
| 25-29 | 30 | 54 | 75 | 329 |
| 30-34 | 20 | 48 | 57 | 177 |
| 35-39 | 11 | 61 | 21 | 107 |
| 40-44 | 10 | 37 | 22 | 68 |
| 45-49 | 6 | 34 | 11 | 42 |
| 50+ | 8 | 49 | 19 | 68 |
| **Total:** | **1025** | **1797** | **2901** | **4394** |

**Supplemental Digital Content 4: Input variables for the base case**

|  | ***Base Case Estimate*** | ***Source*** |
| --- | --- | --- |
| ***Cost estimates*** | | |
| Start-up | See Table 1 | Primary Data Collection |
| Capital | See Table 1 | Primary Data Collection |
| Recurrent | See Table 1 | Primary Data Collection |
| Discount rate | 0.03 | Assumption |
| Yearly ART delivery costs | $515 | ([Kripke, Perales et al. 2016](#_ENREF_6)) |
| ***HIV Infections Averted Calculations*** | | |
| VMMC effectiveness | 0.6 | ([Auvert, Taljaard et al. 2005](#_ENREF_1), [Bailey, Moses et al. 2007](#_ENREF_2), [Gray, Kigozi et al. 2007](#_ENREF_4)) |
| Regional incidence Tabora | See data by age group in source | ([Brown, Grassly et al. 2006](#_ENREF_3), [Stover, Andreev et al. 2014](#_ENREF_9)) |
| Regional incidence Njombe | See data by age group in source | ([Brown, Grassly et al. 2006](#_ENREF_3), [Stover, Andreev et al. 2014](#_ENREF_9)) |
| Ratio of infections averted among females to males | 0.54 |  |
| Average number of years from infection to ART initiation | 1 | Assumption |
| Time horizon | 15 years | Assumption |
| Discount rate | 0.03 | Assumption |
| ***DALY Calculations*** | | |
| Discount rate | 0.03 | Assumption |
| Disability weight (HIV: symptomatic pre-AIDS) | 0.274 | ([Salomon, Haagsma et al. 2015](#_ENREF_8)) |
| Disability weight (HIV/AIDS receiving ART) | 0.078 | ([Salomon, Haagsma et al. 2015](#_ENREF_8)) |
| Disability weight (AIDS: not receiving ART) | 0.582 | ([Salomon, Haagsma et al. 2015](#_ENREF_8)) |
| Male expectation of total life at time of infection (age) | 69 years | ([World Health Organization 2017](#_ENREF_11)) |
| ART coverage rate | 70% | ([Nkingwa M. 2014](#_ENREF_7)) |
| DALYs averted from one HIV infection averted (access to ART) | 3.26 |  |
| DALYs averted from one HIV infection averted (no access to ART) | 21.2 |  |
| Number of years of life expected following infection without ART | 10 | ([Johnson, Mossong et al. 2013](#_ENREF_5)) |

**Supplemental Digital Content 5: Number of circumcisions, total cluster costs and average cost per circumcisions by cluster**


| **Control** | | | | | | | | | | |
| --- | --- | --- | --- | --- | --- | --- | --- | --- | --- | --- |
| **Region** | Njombe | | | | | Tabora | | | | |
| **Cluster** | N1 | N2 | N3 | N4 | N5 | T1 | T2 | T3 | T4 | T5 |
| **Number of parent sites** | 1 | 1 | 1 | 1 | 1 | 1 | 1 | 1 | 1 | 1 |
| **Number of spin-off sites** | 5 | 7 | 4 | 8 | 5 | 5 | 3 | 4 | 5 | 1 |
| **Number of VMMCs** | 219 | 268 | 169 | 102 | 267 | 951 | 272 | 552 | 622 | 504 |
| **Total costs ($)/cluster** | $ 38,936.57 | $ 42,742.22 | $ 41,416.10 | $ 34,339.39 | $ 38,093.19 | $ 38,854.00 | $ 39,938.46 | $ 39,728.60 | $ 45,538.63 | $ 38,155.77 |
| **Cost per VMMC ($)** | $ 177.79 | $ 159.49 | $ 245.07 | $ 336.66 | $ 142.67 | $ 40.86 | $ 146.83 | $ 71.97 | $ 73.21 | $ 75.71 |
|  | | | | | | | | | | |
|  | | | | | | | | | | |
| **Intervention** | | | | | | | | | | |
| **Region** | Njombe | | | | | Tabora | | | | |
| **Cluster** | N6 | N7 | N8 | N9 | N10 | T6 | T7 | T8 | T9 | T10 |
| **Number of parent sites** | 1 | 1 | 1 | 1 | 1 | 1 | 1 | 1 | 1 | 1 |
| **Number of spin-off sites** | 5 | 2 | 7 | 3 | 7 | 3 | 3 | 1 | 2 | 1 |
| **Number of VMMCs** | 359 | 500 | 436 | 218 | 284 | 1187 | 942 | 480 | 673 | 1112 |
| **Total costs ($)/cluster** | $ 43,958.08 | $ 51,133.94 | $ 49,718.24 | $ 40,086.64 | $ 48,581.78 | $ 56,332.60 | $ 55,519.49 | $ 51,042.60 | $ 50,467.97 | $ 58,677.03 |
| **Cost per VMMC ($)** | $ 122.45 | $ 102.27 | $ 114.03 | $ 183.88 | $ 171.06 | $ 47.46 | $ 58.94 | $ 106.34 | $ 74.99 | $ 52.77 |
